# What’s counted counts: the implications of underrepresentation for the application of epigenetic clocks in diverse populations

**DOI:** 10.1186/s13148-025-01954-5

**Published:** 2025-10-03

**Authors:** Samuel F. P. Gibbs, Anna P. Pilbrow, Katrina K. Poppe, Nikki J. Earle, Gregory T. Jones, Allamanda F. Faatoese

**Affiliations:** 1 Christchurch Heart Institute, University of Otago Christchurch, 2 Riccarton Ave, Christchurch, 8022 New Zealand; 2 Heart Health Research Group, University of Auckland, 34 Princes Street, Auckland, 1010 New Zealand; 3 Department of Surgical Sciences, University of Otago, 362 Leith Street, Dunedin, 9016 New Zealand

**Keywords:** DNA methylation, Biomarkers, Equity, Epigenetics, Ageing, Underrepresentation, Population-specific biomarkers

## Abstract

DNA methylation (DNAm) has been touted as a potential unified marker of the contributions of both inherited and environmental factors on an individual’s health. Changes in DNAm have been associated with several chronic diseases and mortality, and DNAm risk scores, or epigenetic clocks, have been proposed as metrics to quantify the process of ‘biological ageing’. Unfortunately, research involving epigenetic clocks is not free from the issues faced in other fields of genomic research. Namely, individuals of European ancestry make up the vast majority of epigenetic study participants and it is unclear whether epigenetic clocks will provide equitable benefits when applied in diverse populations. Although some studies have reported variation in DNAm between populations, it can be difficult to identify the mechanisms underlying these differences. This has implications for clinical application of epigenetic clocks. In this review, we discuss epigenetic clocks, missing diversity in epigenetic research and the potential consequences of the latter on the equitable translation of epigenetic clocks to diverse populations.

## Background

DNA methylation (DNAm) is an epigenetic modification that is heritable across cell division but does not alter the underlying genetic sequence [1]. DNAm involves the addition of methyl-3 carbon groups to cytosine bases at cytosine-guanine dinucleotides, or CpG sites [2]. There are approximately 28 million CpG sites in the human genome, and these are enriched in loci known as CpG islands [3]. DNAm is essential for mammalian development, particularly in cell lineage specification. Consequently, DNAm patterns are highly tissue specific [4] and can influence susceptibility to a wide range of diseases (reviewed here [5]). Mechanistically, DNAm at promoter-associated CpG islands is correlated with decreased gene expression [6]. Although it has been reported that DNAm can, in some cases (typically in gene bodies), be positively correlated with gene expression [7]. DNAm also has other roles including altering the 3D-structure of the genome [8].

Although DNAm patterns are established during development [2] some of these patterns are not fixed, changing stochastically with age [9] or being influenced by environmental exposures [10]. Consequently, changes in whole blood DNAm have been associated with a wide range of chronic diseases, ageing and mortality [11, 12] and several DNAm biomarkers show promise for improving clinical risk prediction [13]. This positions DNAm as a unique molecular marker that has the potential to integrate multiple environmental and heritable influences on gene expression and human health.

Before discussing the promising applications of DNAm as a molecular marker of human health and disease, some key considerations regarding the current state of epigenetic research must be taken into account. First and foremost, there is a pressing lack of diversity, with a widespread and systemic underrepresentation of minority populations. This phenomenon is known as ‘missing diversity’ [14] where the western hegemony of scientific research has resulted in reference genomes and genomic data predominantly representing individuals of European ancestry, who, as of 2018, made up nearly 80% of genome-wide association study participants despite representing only 16% of the world’s population [15, 16]. This issue has been similarly documented in epigenetic research where most epigenome-wide association study participants [17, 18] and the majority of populations used to train major epigenetic clocks [19], are of European ancestry. Epigenetic clocks use DNAm at CpG sites from throughout the genome to predict age or aspects of the ageing process [20]. Predicted epigenetic age has been linked to mortality [12] and risk of several chronic diseases and has potential to improve clinical risk prediction [13]. It has been observed that polygenic risk scores trained in European populations are more likely to produce inaccurate results when applied to non-European populations [15], but it is unclear as to the extent to which this holds true for epigenetic clocks. This review aims to dissect the challenges facing the translation of epigenetic clocks for underrepresented populations by first discussing epigenetic clocks, the potential sources of interpopulation variability in DNAm and how, if unaccounted for, the ongoing underrepresentation of minority populations could lead to the inequitable application of epigenetic clocks to diverse populations in future.

## Main

### First and second generation epigenetic clocks

As an individual ages, their risk of several chronic diseases and mortality increases considerably [21]. However, chronological age does not fully capture the underlying complexity of the ageing process [22] and there is a need for biomarkers of ageing that are better predictors of age-related outcomes than chronological age itself [23]. Early epigenetic research identified altered patterns of DNAm associated with ageing [9, 24, 25] and, in 2011, Bocklandt et al. [26] leveraged this to create an 88 CpG site predictor of chronological age in saliva. In 2013, Horvath et al. published a more comprehensive pan-tissue, 353 CpG site predictor of chronological age (derived from publicly available training and validation datasets) [27], while in the same year Hannum et al*.* published a 77 CpG site blood-based predictor of chronological age [28]. Both predictors exhibit excellent correlations with ageing in external datasets made up of individuals of European, African, and Hispanic ancestry [29] and have been applied in many different populations, as will be discussed later in this review. As the earliest examples of DNA methylation being used to predict age, these are now known as first generation epigenetic clocks.

These first generation clocks can measure epigenetic age acceleration, a surrogate marker for whether an individual is epigenetically younger, or older, than their chronological age. Notably, age acceleration is associated with all-cause mortality [11], and this finding has been validated in over 13,000 individuals across multiple ethnically diverse cohorts (including those of African American, White and Hispanic ethnicity) [12] providing evidence for the hypothesis that epigenetic clocks capture unmeasured aspects of the ageing process [20].

One of the limitations of epigenetic age acceleration estimated from first generation epigenetic clocks is that the estimate is based on chronological age, which is itself an imperfect measurement of ageing. The limitations of this approach were encapsulated by Zhang et al. who found that the relationship between epigenetic age acceleration and mortality attenuates as epigenetic clocks are trained on age in increasingly larger samples [30] highlighting the need to calibrate epigenetic clocks to alternative endpoints.

This gave rise to the second generation of epigenetic clocks, which are calibrated to disease and mortality rather than age. The first of these second generation clocks, DNAm PhenoAge, was published in 2018 by Levine and colleagues [31]. This clock predicts not age but an ageing surrogate quantified through biomarker levels in blood and demonstrated improved associations with mortality and age-related disease compared with first generation clocks, while still being largely correlated with age. Subsequently, Lu et al. developed GrimAge [32], which is derived from DNAm surrogates for eight biomarkers of ageing: smoking pack years, and seven plasma proteins (adrenomedullin, cystatin C, leptin, beta-2 microglobulin, growth differentiation factor 15, plasminogen activator inhibitor 1 and tissue inhibitor metalloproteinase 1). In total, the GrimAge metric uses DNAm from 1013 CpG sites. The age acceleration output of GrimAge is associated with a wide range of age-related conditions and outperforms PhenoAge, the Horvath clock and the Hannum clock. An updated version of the clock [33] which accounts for HbA1c and C-reactive protein was released in 2022 and has stronger associations with mortality than the original.

Dunedin Pace of Ageing measured from the Epigenome (DunedinPACE) is another epigenetic clock that presents a vastly different approach to measuring ageing, leveraging a highly unique and well characterised cohort. As such it has been described as a third generation epigenetic clock. This clock was trained on a composite score that measures changes in biomarker levels at ages 26, 32, 38, and 45 in the Dunedin Longitudinal Study cohort [34], as a proxy for organ system decline. Even though the original score was based on changes in biomarker levels over time, the single time-point DNAm surrogate is associated with age-related phenotypes such as grip strength and organ decline in independent cohorts [35].

These second and third generation of epigenetic clocks appear to have the characteristics we would expect from markers of ‘biological ageing’ in that they associate well with age-related phenotypes and outcomes [36, 37]. It is important to note however that there is no gold standard approach to measuring ‘biological ageing’, and so there is no reference point to truly validate these clocks against. This has implications for the application of clocks in diverse populations as we will now discuss.

### The functioning of epigenetic clocks across populations

Based on estimates from first generation epigenetic clocks it has been reported that some populations may have different rates of biological ageing [29, 38]. Epigenetic clocks have now been applied in a wide range of populations and settings including in Asian [39–44], African [45, 46], Southern and Central American [29, 47–51], and Indigenous populations [52–55]. Although these studies largely validate the functioning of epigenetic clocks, we argue that this does not necessarily guarantee that epigenetic clocks will produce accurate results when applied in multi-ethnic contexts. Although GrimAge and PhenoAge were validated in samples made up of European, African American, and Hispanic participants, many other populations fall outside these three groups. This could affect the application of clocks in multi-ethnic settings where factors unrelated to ageing drive differences in clock estimates between populations.

Genetic variation can influence DNAm through a variety of mechanisms. Single nucleotide polymorphisms (SNPs) that disrupt the CpG site can prevent DNAm outright [56], SNPs within the 50 base pair probe of DNAm array CpG sites can alter measured DNAm levels [57] by influencing hybridisation efficiency, and SNPs beyond the CpG site or probe [58], known as methylation quantitative trait loci (meQTLs) can also affect DNAm. MeQTLs can be further separated into cis-meQTLs or trans-meQTLs based on their proximity to the CpG site. The traditional definition for a trans-meQTL involves a SNP further than one megabase from the affected CpG site. Genome-wide association studies have estimated heritability for epigenetic age acceleration to range from 0.10 to 0.37 depending on the epigenetic clock and study[ 59, 60]. In future, researchers should consider both genetic and epigenetic contributions to epigenetic ageing to facilitate more reliable interpretations of epigenetic clock estimates.

Genetic variants more common in one population compared with another could lead to spurious offsets in clock estimates that, in turn, could be interpreted as genuine (e.g. one population may appear to have higher clock scores than another due to a large proportion of the alternate group carrying a variant, rather than having an accelerated “biological age”). The PhenoAge clock includes CpG sites that are in close proximity to genetic variants more common in some populations than others and may be affected by this issue [61]. Offsets introduced in clock estimates by SNPs more prevalent in test populations compared with training populations are less likely to affect the association between a clock and its target measure, age for instance [46]. For a minority population with a common SNP that prevents DNAm at a CpG site this would likely affect clock estimates at the level of the individual rather than at the level of the population. However, further research is needed to confirm this. It was hypothesised that such confounding can be overcome by adjusting for the individuals specific genotype or excluding potentially polymorphic sites [29]. A 2024 study [46] confirmed this hypothesis finding that epigenetic clock estimates had improved accuracy if cis-meQTL CpG sites were excluded from clock calculation. The presence of such sites could be problematic for populations who are absent from databases of genomic variation [62] and studies that do not have access to genome-wide SNP data.

Although DNAm can be influenced by both inherited and environmental factors, research into the exact contribution of each is lacking. A 2018 study [63] comparing 18 year old monozygotic and dizygotic twin pairs found that, at autosomal CpG sites, most of the variation could be attributed to environmental influences rather than genetic ones, but that the contribution of each differed based on CpG site and genomic position. The study also found that CpGs with known environmental influences (e.g. cg05575921 in the *Aryl-Hydrocarbon Receptor Repressor* (*AHRR)* gene is strongly influenced by exposure to tobacco smoking) had a higher correlation in monozygotic twin pairs who had never smoked cigarettes than dizygotic twin pairs who had never smoked cigarettes. This data suggests that genetic factors are contributing to variability at exposure associated CpG sites without the presence of the exposure (in this case smoking). These findings add a level of nuance to a twin study from 2016 [64], which found that, on average, the CpG sites on the 450 k array had an average heritability (*h*^2^) of 0.19 and at 39% of CpG sites, common SNPs explained < 0.01 of variance suggesting that environmental influences have a much larger contribution to DNAm across the genome.

**Fig. 1:**
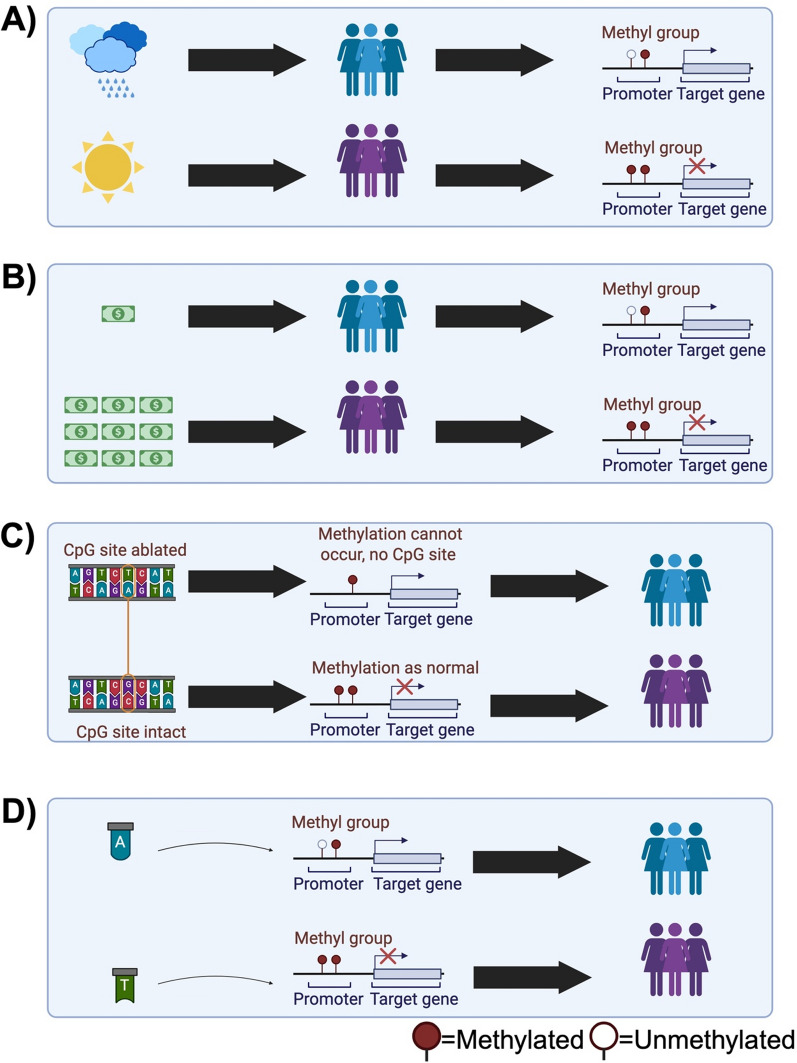
Potential drivers of population differences in DNA methylation for two populations (blue and purple)

To summarise, DNAm differences between populations can be affected by multiple environmental, and genetic factors [58]. These include differences in environment [65, 66] (Fig. 1A), the social determinants of health (e.g. socioeconomic status [67], Fig. 1B), SNPs that disrupt the CpG site [56] (Fig. 1C), SNPs under an array probe [57], and meQTLs (Fig. 1D) [60, 68, 69]. As the above could all vary markedly between ancestral groups there can be difficulty in disentangling differences in epigenetic clock estimates between populations. Further complications include offsets in epigenetic clock outputs introduced by the choice of normalisation schema [70, 71] and offsets in clock estimates based on the array type used [71]. It has been demonstrated that the epigenetic clock estimates limited to the probes contained on the EPIC v2 array produce inflated estimates of age using Hannum’s clock (by over 20 years, on average) [72]. We note that the resulting output was still correlated with age, so the residual age acceleration estimate should be consistent. Age acceleration calculated with the absolute difference will be different, however. Missing probes can be “imputed” using the average values from training data, or open source datasets [29]. This effectively shifts the output to be closer to that of the training population. Normalisation schema, array type, how age acceleration was measured, and imputation strategy are all factors researchers should be aware of when applying epigenetic clocks in diverse contexts.

An observed difference in DNAm levels between populations could be driven by (A) differential environmental exposures, (B) differential exposure to social disadvantage, e.g. lower socioeconomic status, or reduced access to healthcare, (C) genetic variants that change the CpG dinucleotide sequence, thus preventing DNAm, or (D) meQTLs with diverging allele frequencies. Dark circles indicate a methylated CpG site, empty circles indicate an unmethylated CpG site. No circle indicates absence of a CpG site.

Applying and validating epigenetic clocks in more diverse populations is necessary for the validation of their use as ageing biomarkers [73]. Several studies have compared epigenetic ageing in mixed populations (summarised in Table 1). In general, clocks appear robust across the groups tested in terms of association with outcomes such as mortality [12, 31–33, 74, 75]. One common observation with second generation clocks was Black Americans appearing to have accelerated ageing when compared with White Americans [31–33, 67], even in the absence of social disadvantage such as access to education [76, 77] and poverty status [78]. Notably, the difference between Black and White Americans has only been observed for second generation clocks; age acceleration estimates derived from first generation clocks typically show decreased age estimates for Black Americans compared to White Americans [29, 76, 79]. How can we be sure which observation, if any, is correct? We suggest caution when interpreting differences in epigenetic clock estimates levels between populations and differences in epigenetic age acceleration until we understand the true drivers of any observed differences.

**Table 1:** Review of studies comparing epigenetic clock estimates between racial/ethnic groups

Citation	Clock(s)	Array	Populations	Tissue	Summary
Maunakea et al. [52]	DunedinPACE	EPIC	144 Native Hawai’ian, 113 Japanese American, and 119 White American participants	PBMCs enriched for monocytes	Native Hawai’ians exhibited highest proportion of participants with advanced ageing. Differential associations between DunedinPACE and lifestyle variables were observed between ethnicities
Yusipov et al. [ 38]	12 clocks including PC clocks [80] and DunedinPACE	450k, EPIC	Samples from 25 countries (predominantly European but with representation of Asian, African, and Hispanic participants within dataset)	10 tissues, including blood	Authors measured age acceleration using absolute difference between predicted age and chronological age. They observed a high degree of discordance in epigenetic age acceleration estimates even within populations
Freilich et al. [81] 2024	Hannum, Horvath, PhenoAge, GrimAge, DunedinPoAm	EPIC	3000 White American and 674 Black American participants	Whole blood	Black participants had higher GrimAge acceleration and DunedinPoAm, but lower Hannum acceleration compared to White participants
Harris et al. [ 82]	Horvath, PhenoAge, GrimAge, DunedinPACE	EPIC	17 Indigenous American, 195 Asian American, 811 Black American, 435 Hispanic American and 2746 White American participants	Venous blood	Black participants had higher GrimAgeAA and DunedinPACE compared to White participants Asian participants had lower PhenoAgeAA and GrimAgeAA Hispanic participants had lower GrimAgeAA, but higher DunedinPACE compared with White participants
Chen et al. [ 83]	PhenoAgeAA	EPIC	1476 White American and 230 Black American participants	Blood	Black participants had higher PhenoAgeAA compared to White participants when comparing participants who did not finish high school to college graduates
Chang et al. [ 84]	PhenoAge, GrimAge, DunedinPACE	450k, EPIC	667 Black American women and 3691 White American women	Blood	Black women had higher GrimAgeAA and DunedinPACE, but lower PhenoAgeAA compared with White women
Forrester et al. [85]	IEAA, EEAA, PhenoAgeAA GrimAgeAA	EPIC	380 Black American and 543 White American participants	Whole blood	White men had highest EEAA, Black men had highest GrimAgeAA and Black women had highest PhenoAgeAA
Mutambudzi et al. [ 86]	PhenoAge, GrimAge	Not reported	435 Black American, 351 Hispanic American and 2107 White American participants	Blood	Black participants had higher GrimAgeAA relative to White and Hispanic participants
Crimmins et al. [87]	Horvath, Hannum, PhenoAge, GrimAge, DunedinPACE, GrimAge2, CausAge [88], AdaptAge [88], DamAge [88]	EPIC	16% Black American, 14% Hispanic American and 66% White American participants. Total = 4101	Blood	Report variable associations between epigenetic clocks and race and ethnicity
Jain et al. [ 89]	Horvath, Hannum, PhenoAge, GrimAge	450k	343 Black American, 154 Hispanic American and 1374 White American participants	Blood	Black women overrepresented in highest quartile of PhenoAgeAA relative to White and Hispanic participants
Iannuzzi et al. [ 50]	Horvath, EEAA, IEAA, Hannum, Skin and blood [90], PhenoAge, GrimAge	EPIC	24 Wichi and 24 Crillios participants	Whole blood	Wichi participants had higher EEAA, HannumAA, PhenoAgeAA, GrimAgeAA compared with Crillios participants
Korous et al. [ 77]	10 including Horvath, Hannum, Bocklandt, PhenoAge, DunedinPoAm	EPIC	10.10% Black American, 9.02% Hispanic American and 77.5% White American participants Total n = 4104	Blood	Variable levels of accelerated ageing across White, Hispanic and Black participants depending on parental education
Kim et al. [ 91]	IEAA, EEAA, PhenoAge, GrimAge, DunedinPACE	EPIC	319 Black American and 576 White American participants at year 15	Whole blood	PhenoAgeAA was associated with adverse childhood experience in Black but not White participants
Bozack et al. [ 92]	Horvath, Skin and Blood	450k	62 Black American, 16 Asian American, 25 Hispanic American and 325 White American participants	Cord blood	Black participants had higher Skin and blood AA compared with White participants
Shen et al. [ 78]	DunedinPACE	EPIC	237 Black American and 233 White American participants	PBMCs	Black participants have higher DunedinPACE compared with White participants
Faul et al. [ 37]	13, including Horvath, Hannum, PhenoAge, GrimAge as well as PC clocks	EPIC	9.9% Black American, 8.9% Hispanic American and 81.3% White American participants Total = 3581	Blood	Black and Hispanic participants have decelerated or accelerated ageing dependent on clock, when compared with White participants. PC trained clocks alter effect sizes, but direction of effect is consistent
Lu et al. [ 33] (GrimAge 2)	GrimAge, GrimAge 2	EPIC, 450k	Trained on White American participant and Validated across White American, White European, Black American and Hispanic American participants	Blood	GrimAge 2 AA is generally higher in Black compared with White and Hispanic participants tested, but association with mortality is consistent
Elliott et al. [ 93]	EEAA, IEAA, PhenoAge, Skin and blood	450k, EPIC	400 South Asian and 400 European participants; 444 White British and 472 Asian Pakistani participants across two cohorts	Peripheral blood	South Asian participants had higher PhenoAgeAA after adjustment for lifestyle factors. Difference in other clocks attenuated after adjustment
Joyce et al. [ 48]	Hannum	450, EPIC	Multiple cohorts with White American, Black American and Hispanic American participants	Blood, Buccal, Cord blood, Peripheral blood, Leukocytes	Associations between HannumAA and SES were largely consistent across race and ethnicity
Schmitz et al. [ 94]	Eight, including Horvath, Hannum, PhenoAge, GrimAge, and DunedinPoAm	450k, EPIC	901 Black American, 122 Hispanic American and 3251 White American participants across two cohorts	Monocytes, Blood	Associations between age acceleration and SES are largely consistent across ethnicities
Graf et al. [ 95]	Horvath, Hannum, PhenoAge, GrimAge, DunedinPoAm	EPIC	75% White American and 17% Black American participants Total = 3928	Blood	Black participants had higher rates of GrimAgeAA and DunedinPoAm when compared with White participants but not for other clocks
Roberts et al.[ 96]	Horvath, Hannum, PhenoAge, GrimAge	450k	2840 Black American and 2760 White American participants across three cohorts	Whole blood	Differential associations across cohort (ARIC has all Black, FHS has all white participants) and incident atrial fibriliation based on clock used
Joyce et al. [ 74]	IEAA, EEAA, GrimAge	450k, EPIC	424 Black American, and 2724 White American participants across two cohorts	Whole blood	Found no evidence for race specific association between epigenetic age acceleration and cardiovascular health
Matías-García et al. [75]	Hannum, EEAA, Horvath, IEAA, PhenoAge, GrimAge	450k, EPIC,	European, Hispanic and African American participants across seven cohorts	Whole blood	Consistent associations between epigenetic age acceleration and kidney health across populations
Crimmins et al. [ 67]	13, including Horvath, Hannum, PhenoAge, GrimAge, DunedinPoAm	EPIC	10% Black American, 9% Hispanic American and 81% White American participants. Total = 3966	Whole blood	Black and Hispanic participants had variable age acceleration compared with White participants depending on clock used
Tajuddin et al. [ 97]	Horvath, IEAA, EEAA	EPIC	244 African American and 243 White American participants	Blood	EEAA was lower in African American participants compared with White participants
Liu et al. [ 76]	PhenoAge	450k	865 White American, 605 Black American and 364 Hispanic women	Blood	Found that PhenoAgeAA mediates some of mortality difference between Black and White participants. Similar was observed when comparing Hispanic with White participants
Lu et al. [ 32] (GrimAge)	GrimAge, IEAA, EEAA, PhenoAge	450k, EPIC	Trained on White participants and Validated across White American, Black American and Hispanic participants	Blood	Median GrimAgeAA was positive for all Black participants and negative for all White and Hispanic participants except Black participants in Jackson Heart Study
Levine et al. [31] (PhenoAge)	PhenoAge, Horvath, Hannum	450k, EPIC	Trained in Italian cohort with 456 participants, validated in five cohorts with White American, Black American and Hispanic American participants	Blood	PhenoAge was highest in Black participants, then Hispanic participants and lowest in White participants. Predicts mortality consistently across groups
Quach et al. [79]	IEAA, EEAA	450k	1277 Black American, 140 Asian American, 2196 White American and 456 participants from Italian cohort	Blood	Of the populations compared, EEAA was only lower in Asians relative to other populations
Chen et al. [ 12]	IEAA, EEAA, Hannum, Horvath	450k	13,089 Black American, White American, White European, or Hispanic American participants	Blood, Saliva, Breast	Direction of association of age acceleration measures with time to death was consistent across all populations
Horvath et al. [ 29]	IEAA, EEAA, Horvath, Hannum	450k	1387 participants of African ancestry, 2932 White participants, 657 Hispanic participants, 127 East Asian participants, and 59 Tsimane participants	Blood, saliva, brain	Epigenetic clocks were similarly correlated with epigenetic age across ethnicities, but there were differences in age acceleration between populations compared

Abbreviations used in this table: *PBMC* peripheral blood mononuclear cells, *AA* age acceleration, *IEAA* intrinsic epigenetic age acceleration, *EEAA* extrinsic epigenetic age acceleration, *DunedinPoAm* dunedin pace of ageing methylation

### Further considerations for epigenetic clocks with underrepresented populations

One study [70] has recommended that epigenetic clocks be interpreted at a population level arguing that the rank of an individual’s epigenetic age relative to the population is more robust to sources of technical variation, like normalisation schema. This raises important questions regarding the application of epigenetic clocks at an individual level and whether epigenetic clock estimates can even be interpreted on the level of the individual. As discussed, epigenetic age acceleration can be estimated either by taking the absolute difference between age and chronological age, or the residual of predicted age regressed on chronological age. Estimating age acceleration via the absolute difference will be more feasible in a point of care setting (as the residual requires data from many individuals to generate an average), but this is where offsets (caused by technical artefacts alluded to above) could become a major source of inequity. We argue that observed epigenetic age acceleration in an individual from a marginalised group could plausibly be attributed to inequity experienced by members of marginalised groups, as has been observed previously [78]. To be interpreted at an individual level epigenetic clocks will need to be better calibrated, and more robust to potential sources of confounding including technical variation or SNPs. Standalone epigenetic clocks such as DunedinPACE that do not require calibration to real world variables such as age could provide a solution to this issue [38], and it appears that epigenetic clocks trained on principal components may provide more robust estimates [37, 80]. Training clocks in more diverse, more representative cohorts will also improve calibration [19].

Other considerations for applying epigenetic clocks in underrepresented populations include data sovereignty, the importance of open source epigenetic clocks, and the interpretation of epigenetic clock outputs. Data sovereignty is about maintaining control over information arising from participants and is particularly important for populations who are underrepresented in research such as Indigenous Peoples as it provides an avenue through which greater autonomy within research, and greater control over research outcomes, can be obtained [98]. The need for greater control is best demonstrated by past instances of research harm, which have deterred Indigenous participation in research [99]. Examples include the non-consensual use of Havasupai Tribe tissue samples [100] and Warrior Gene saga [101], where a genetic variant was falsely reported as being associated with violence and antisocial behaviour in the New Zealand Māori population. Both incidents involved researchers violating the participants control over research samples and we argue that the lessons learned are highly relevant for researchers conducting work with epigenetic clocks in underrepresented populations. Practical suggestions for enabling data sovereignty include keeping epigenetic clocks open-source and empowering underrepresented populations to carry out work with epigenetic clocks in-house. The latter is highly feasible as the majority of epigenetic clocks are open source so it is imperative that they remain this way. For clocks that are not open source, the need for data to be potentially submitted to a private platform may be viewed with scepticism by underrepresented communities and provide a deterrent for participating in epigenetic research.

For epigenetic clocks to be used as clinical trial endpoint markers of biological ageing, it will be necessary to establish epigenetic ageing as modifiable by intervention [102]. Demonstrating this will have implications for the framing of perceived differences in epigenetic age between racial or ethnic groups. One review found that epigenetic clock research can include race or ethnicity without adequately discussing these concepts as surrogates for other sociocultural determinants of health [103]. Race and ethnicity are social constructs with race being based on perceived physical differences and ethnicity referring to groupings based on shared culture. These categorisations are not fixed and our perceptions of what physical characteristics define racial categories can change with time. Importantly, race or ethnicity do not refer to some innate biological categories [104]. To put this in context, reporting an observation that one racial group has increased epigenetic age when compared with another whilst ignoring the broader social determinants of health poses the risk of framing race as “biologically acquired” [105]. Although health inequity experienced by some minority populations could foreseeably result in a signal of “accelerated biological ageing”, there are many other factors that could be confounding this observation, as we have discussed already. It is imperative that we understand the drivers of epigenetic clock estimates and establish that epigenetic ageing is not fixed to minimise the potential for harm when interpreting differences in epigenetic age between populations.

## Conclusions

The functional importance and dynamic nature of DNAm suggest a strong potential as a marker of health and disease. Epigenetic clocks show immense potential as tools to improve risk prediction and risk monitoring for a host of chronic diseases associated with ageing. As discussed, epigenetic clocks have been applied in several different populations and appear to capture age associated outcomes well across different groups. However, a lack of understanding of the factors that drive variation in DNAm across populations and how this might impact epigenetic clocks is a major gap. Before attempts at clinical translation of DNAm-based risk tools, it is essential that the factors that drive interpopulation variation in DNAm are accounted for. Clinically implementing genetically influenced epigenetic clocks in diverse populations could lead to inaccurate estimates and potentially exacerbate the health disparities experienced by some minority populations. To avoid this, increased representation of minority populations in epigenetic research and in the training samples for epigenetic clocks is critical. We conclude with three recommendations for improving the equity of epigenetic clock research for underrepresented populations: (1) improving the reporting of factors that may drive interpopulation differences in diverse studies, (2) improving assessment of CpG sites for genetic variants that may introduce spurious offsets in non-training populations, and (3) expanding the diversity of epigenetic research through consortia or the inclusion of more ethnicities in epigenetic clock training and validation populations.

## Data Availability

Not applicable.

## Abbreviations

DNAm: DNA methylation
CpG: Cytosine-guanine dinucleotide
MeQTL: Methylation quantitative trait loci
DunedinPACE: Dunedin pace of ageing measured from the epigenome
